# ITRAQ and PRM-based quantitative saliva proteomics in gastric cancer: biomarker discovery

**DOI:** 10.3389/fmolb.2025.1640508

**Published:** 2025-10-14

**Authors:** Zhanyan Liu, Jieren Liu, Zaid Chachar, Jimao Mo, Haoran Chi, Runtao Wen, Kaixin Luo, Lei Huang, Guanlin Li, Chenhao Zhang, Yuanru Mao, Yuanzhe Cai, Zhengzhi Wu, Feijuan Huang

**Affiliations:** ^1^ The First Affiliated Hospital of Shenzhen University, Shenzhen Second People's Hospital, Medical Innovation Technology Transformation Center of Shenzhen Second People's Hospital, Shenzhen University, Shenzhen Translational Medicine Institute, Shenzhen, China; ^2^ Changsha Hospital of Traditional Chinese Medicine Affiliated to Hunan University of Traditional Chinese Medicine, Changsha, Hunan, China; ^3^ School of Big Data and Internet, Shenzhen Technology University, Shenzhen, China; ^4^ School of Data Science, The Chinese University of Hong Kong, Shenzhen, China; ^5^ Shenzhen Wemed Medical Equipment Co., Ltd., Longgang District, Shenzhen, China

**Keywords:** iTRAQ, PRM, proteomic, gastric cancer, saliva, biomarkers

## Abstract

**Background:**

Salivary proteomics is a non-invasive, low-cost, and real-time diagnostic approach increasingly applied in cancer research. Salivary biomarkers hold particular promise for the early identification of gastric cancer (GC). This study aimed to detect salivary proteins altered in GC and evaluate their potential as novel non-invasive biomarkers.

**Methods:**

We analyzed salivary proteomes from GC patients (group 1: *n =* 12; group 2: *n =* 13) and healthy controls (*n =* 11) using isobaric tags for relative and absolute quantitation (iTRAQ). Differentially expressed proteins (DEPs) were identified and functionally annotated using gene ontology (GO), Kyoto Encyclopedia of Genes and Genomes (KEGG) analysis, and protein–protein interaction (PPI) networks. Candidate proteins were further validated by parallel reaction monitoring (PRM), and prognostic significance was assessed through Kaplan–Meier (KM) survival analysis.

**Results:**

A total of 671 proteins with unique peptide segments were identified. Among them, 124 and 102 proteins were significantly differentially expressed in GC groups 1 and 2, respectively, compared with controls. Fifty-six overlapping DEPs were detected between the two GC groups, including 24 upregulated and 32 downregulated proteins. Functional enrichment and PRM validation highlighted four key proteins (S100A8, S100A9, CST4, CST5) with consistent differential expression. Interestingly, CST4 and CST5 were downregulated in saliva but upregulated in GC tissue and blood.

**Conclusion:**

Our findings demonstrate that salivary proteins, particularly S100A8, S100A9, CST4, and CST5, hold significant potential as non-invasive biomarkers for gastric cancer detection. These results provide new insights into saliva-based diagnostics and highlight the importance of cross-comparison with tissue and blood expression profiles.

## 1 Introduction

Gastric cancer (GC) is a major global health issue, with more than 1 million new cases diagnosed worldwide in 2020, representing 5.6% of all cancer cases diagnosed. The epidemiological trends of GC vary significantly among regions. Eastern Asia (Japan, Korea, and Mongolia) and Eastern Europe had the highest incidence rates (Sung et al., 2021). Although treatment efficacy has improved over the past 10 years, the overall survival remains low (Zong et al., 2016; Du and Li, 2020). The past 5-year research data of the United States highlighted the low survival rate for patients diagnosed with GC compared to all cancers (Thrift and El-Serag, 2020). More than 40% of new cases and deaths of GC occur in China. GC is the third-most common cancer in China; 396,500 new cases of GC were diagnosed in China in 2016, and the crude incidence is 28.68/10^5^ (Chen et al., 2016; Zheng et al., 2022). GC is the main cause of cancer-related deaths in China, and the 5-year survival rates of GC are low. It is estimated that approximately 288,500 Chinese people died from GC in 2016. In 2022, an estimated 358,700 new cases of gastric cancer were diagnosed in China, ranking it among the top five leading cancer types, with approximately 260,400 deaths reported in the same year (Xie et al., 2025). Because more than 80% of patients are diagnosed at an advanced stage, the prognosis is poor, and treatment options are limited. In addition, the incidence is increasing among young adults, and younger people usually have a worse prognosis due to signet ring cell carcinoma and poorly differentiated malignancy. They also frequently appear with a more advanced form of the disease than older patients (Wong et al., 2021; Parsanathan et al., 2024). Therefore, GC screening should be prioritized in clinical recommendations and government agendas to reduce related morbidity and mortality, particularly among younger populations.

Saliva is an emerging diagnostic fluid that contains a wide range of molecular biomarkers, including proteins, nucleic acids, and metabolites that reflect both local and systemic physiological states (Jiao et al., 2025; Yousif et al., 2025). While traditionally used in oral health studies, recent research has demonstrated that systemic diseases such as breast, pancreatic, and lung cancers can also alter the salivary proteome through mechanisms such as exosome-mediated transfer, systemic inflammation, and metabolic reprogramming (Kaczor-Urbanowicz et al., 2019; To et al., 2019). These findings support the notion that cancer-related signals originating from distant organs, including the stomach, can be detected in saliva.

Saliva collection is simple, noninvasive, and cost-effective, making it ideal for population-level screening, particularly in low-resource settings. Building on these advantages, this study explores the potential of salivary proteomics to uncover novel biomarkers for early detection of gastric cancer (Picolo et al., 2024; Surdu et al., 2025).

Current diagnostic methods, including upper endoscopy and conventional tumor biomarkers (e.g., CEA and CA19-9), are invasive, expensive, or lack sensitivity and specificity for early-stage detection. Therefore, there is a critical need for novel, noninvasive diagnostic approaches that can facilitate the early identification of GC. Saliva, a readily accessible and non-invasively collected biofluid, contains a rich repertoire of proteins and has shown promise in cancer biomarker research. In this study, we investigated the association between the salivary proteome and gastric cancer (GC), hypothesizing that specific salivary protein profiles may serve as diagnostic biomarkers. Using iTRAQ- and PRM-based proteomic analyses, we aim to identify and validate differentially expressed salivary proteins with potential diagnostic value for GC.

## 2 Materials and methods

### 2.1 Sample source

Saliva samples were collected from GC patients and healthy controls in Shenzhen, Guangdong, China, from September 2019 to September 2020.

Inclusion criteria for GC patients included the following: (1) histologically confirmed gastric cancer; (2) no prior chemotherapy or radiotherapy; (3) age between 18 and 75 years; (4) ability to provide informed consent.

Inclusion criteria for non-GC controls: (1) no current or previous diagnosis of malignancy; (2) no major systemic illness or acute infection; (3) matched for age and sex as far as possible.

Exclusion criteria for all participants included: active oral diseases (e.g., gingivitis and periodontitis), autoimmune conditions, chronic inflammatory disorders, or recent (within 3 months) use of antibiotics, steroids, or immunosuppressants.

All participants provided informed written consent, and the study protocol was approved by the ethics committee of the First Affiliated Hospital of Shenzhen University (Approval No. KS20190418003). Tables 1 and 2 list the clinical sample information in this experiment. Unstimulated saliva was collected into sterile plastic tubes, and then the saliva tubes were centrifuged at 10,000 rpm for 10 min at −4 °C. Separate aliquots of supernatants were stored frozen at −80 °C until analysis. Tables 1 and 2 summarize the overall clinical sample groups involved in both the biomarker discovery (iTRAQ) and validation (PRM) phases of the study.

**TABLE 1:** Demographic information of the participants in this study (*n =* 68).

		Biomarker discovery phase iTRAQ			Biomarker validation phase PRM	
Demographic variable		GC 1 12	GC 2 13	Non-GC 11	GC 16	Non-GC 16
Age, years		56.35 ± 3.04	52.45 ± 4.96	55.33 ± 6.78	56.2 ± 9.10	54.8 ± 10.4
Gender	Male	8	7	5	10	8
	Female	4	5	6	6	8

**TABLE 2:** Overlapping differentially expressed protein profiles.

Uniprot ID	Protein name	Score	Coverage	Unique peptides	GC1		GC2	
					FC	-Log10*P*	FC	-Log10*P*
P15515	HTN1	54.380	28.070	1.000	0.203	2.091	0.267	1.980
P02808	STATH	47.310	48.390	1.000	0.235	1.446	0.260	1.409
P23219	PTGS1	25.180	2.340	1.000	0.259	2.345	0.300	2.238
Q04118	PRB3	395.670	3.240	1.000	0.291	6.914	0.504	4.750
Q8TAX7	MUC7	40.930	9.810	5.000	0.319	5.728	0.309	5.257
Q05315	CLC	45.780	25.350	3.000	0.325	4.392	0.470	5.513
P68032	ACTC1	721.310	38.200	3.000	0.346	2.749	0.561	2.002
P28325	CST5	243.860	49.300	5.000	0.361	6.273	0.626	4.451
Q96DR5	BPIFA2	234.630	47.790	12.000	0.365	5.264	0.442	4.272
P23280	CA6	1667.150	64.940	13.000	0.379	5.046	0.389	5.190
P01036	CST4	7066.280	68.790	5.000	0.408	2.673	0.656	1.691
P80303	NUCB2	181.320	32.380	12.000	0.425	6.775	0.478	5.460
P0C0S5	H2AZ1	58.670	12.500	1.000	0.459	3.813	0.415	3.558
P07711	CTSL	52.770	16.520	4.000	0.463	3.050	0.409	3.490
P41218	MNDA	29.050	4.180	1.000	0.476	1.965	0.562	1.790
P08697	SERPINF2	47.710	4.280	2.000	0.492	3.712	0.540	3.450
P04745	AMY1	38,226.830	75.930	6.000	0.508	2.519	0.558	2.146
Q8N4F0	BPIFB2	450.570	29.040	10.000	0.538	4.377	0.579	4.215
Q96DA0	ZG16B	1072.930	48.080	7.000	0.551	5.945	0.409	7.574
P16870	CPE	42.110	11.340	5.000	0.562	7.112	0.535	8.202
Q15782	CHI3L2	38.390	28.460	8.000	0.563	5.034	0.615	4.361
Q8IUE6	H2AC21	20.210	12.310	1.000	0.576	2.731	0.416	4.355
P30408	TM4SF1	69.450	4.460	1.000	0.586	1.763	0.384	2.500
P06733	ENO1	2199.190	63.590	17.000	0.605	4.096	0.630	3.865
Q9Y287	ITM2B	35.470	4.890	1.000	0.605	2.267	0.225	3.737
P58499	FAM3B	33.210	23.830	5.000	0.613	4.271	0.454	5.139
Q8NBJ4	GOLM1	90.060	22.690	9.000	0.616	4.328	0.630	4.427
Q8NBS9	TXNDC5	27.500	4.170	2.000	0.633	3.184	0.508	1.791
Q9HC38	GLOD4	28.200	20.770	7.000	0.652	2.437	0.622	2.763
P68431	H3C1	43.340	22.060	4.000	0.661	2.922	0.589	5.211
Q01813	PFKP	36.910	3.570	1.000	0.662	1.335	0.262	5.694
P30740	SERPINB1	878.060	51.720	20.000	0.663	4.894	0.663	5.400
Q8NFT8	DNER	58.450	8.410	5.000	1.504	2.849	1.665	6.735
P01699	IGLV1-44	32.070	14.410	2.000	1.519	1.347	1.673	1.470
O43617	TRAPPC3	20.550	4.440	1.000	1.542	1.879	1.685	6.997
P59665	DEFA1	186.450	20.210	4.000	1.594	1.343	2.215	3.253
P80188	LCN2	197.050	58.080	10.000	1.653	5.050	1.790	4.333
P35321	SPRR1A	70.530	87.640	2.000	1.784	4.842	1.802	3.334
P13987	CD59	47.200	25.000	3.000	1.818	2.814	2.032	4.141
P01764	IGHV3-23	175.780	41.030	2.000	1.827	1.380	1.674	1.871
P01773	IGHV3-30	28.830	15.970	2.000	1.832	3.427	1.774	2.831
P47755	CAPZA2	60.380	6.590	3.000	1.855	3.712	1.705	3.604
P22528	SPRR1B	42.940	70.790	1.000	1.957	3.746	2.098	4.044
P19013	KRT4	1157.030	55.990	29.000	1.988	6.867	1.558	6.346
O43847	NRDC	30.570	0.960	1.000	2.085	4.165	1.836	3.381
Q53FA7	TP53I3	33.670	6.020	2.000	2.090	4.682	2.032	4.459
P31151	S100A7	310.280	68.320	5.000	2.110	2.796	1.745	4.218
Q07654	TFF3	129.550	22.500	2.000	2.225	3.473	1.553	3.404
P06702	S100A9	3348.350	82.460	9.000	2.289	5.339	2.418	6.639
P08246	ELANE	204.640	36.330	6.000	2.299	5.164	1.613	3.283
P01040	CSTA	141.150	75.510	6.000	2.340	7.692	1.595	4.979
P13646	KRT13	1133.620	50.220	17.000	2.343	4.497	1.529	6.213
P05109	S100A8	3608.280	94.620	14.000	2.573	4.888	1.962	6.975
Q86SG5	S100A7A	207.240	47.520	1.000	2.698	4.758	2.135	5.500
P02538	KRT6A	1166.900	44.150	1.000	2.936	5.146	1.519	3.991
Q14210	LY6D	53.140	23.440	3.000	3.104	2.277	1.858	2.794

No formal power or sample size calculation was performed due to the exploratory nature of this pilot proteomic study. The number of samples (*n =* 68 total; 36 for discovery, 32 for validation) reflects practical constraints in obtaining high-quality, clinically annotated saliva samples. We acknowledge this as a limitation and recommend larger-scale validation in future studies.

### 2.2 Identification and quantification of saliva proteomics by iTRAQ experiment

These samples were routinely processed for iTRAQ experiments. According to Noto et al. (2019), peptides from each group were labeled with the following tags: 117 and 118 tags for GC 1, 119 and 121 tags for GC 2, and 113 and 114 tags for NC, respectively. The peptides were dissolved in 0.5 M TEAB and tagged in accordance with the iTRAQ kit’s instructions as follows: The peptides and the labeled reagent were combined, and the mixture was incubated for 2 h at room temperature, followed by desalting and vacuum-drying of the labeled peptides. The raw data were processed using Proteome Discover 1.3 (Thermo Fisher Scientific, v.1.3) (Chen et al., 2020). Differentially expressed proteins (DEPs) were identified using a threshold of p < 0.05 and fold change >1.5 or <0.67. These thresholds are consistent with widely accepted standards in quantitative proteomics, designed to balance statistical significance with biological relevance, especially in small discovery cohorts. A two-tailed Student’s t-test was applied to compare GC and non-GC groups.

### 2.3 Bioinformatics analysis of the DEPs

Differentially expressed proteins (DEPs) were first input into the STRING database (https://cn.string-db.org/) to construct a protein–protein interaction (PPI) network, which was visualized using Cytoscape (version 3.9.0). To functionally characterize the DEPs, we performed Gene Ontology (GO) and Kyoto Encyclopedia of Genes and Genomes (KEGG) pathway enrichment analyses using the clusterProfiler package in R. GO analysis categorized the proteins into three main domains: biological processes (BP), molecular functions (MF), and cellular components (CC). For statistical significance, we applied a Bonferroni-adjusted p-value of <0.05 for GO terms and a p-value of <0.05 for KEGG pathway enrichment analysis.

### 2.4 LC-PRM analysis

Ten key DEPs were verified by PRM. Two pooled samples were prepared for PRM using the peptides. The peptides were subjected to an NSI source, tandem mass spectrometry (MS/MS), in Q ExactiveTM Plus (Thermo), connected online to the UPLC, according to Scheltema et al. (2014). The fragments were then detected in the Orbitrap at a resolution of 17,500 after the peptides were selected for MS/MS with an NCE setting of 27.20. MS/MS scans were followed by one MS scan using a data-independent technique. The AGC settings for complete MS and MS/MS were 3E6 and 1E5, respectively. The maximum IT for full MS and auto MS/MS was set at 20 ms. The MS/MS isolation window was set to 2.0 m/z.

The MS data generated were processed using Skyline software (version 3.6) (Adams et al., 2020). Peptide settings: Trypsin Max missed cleavage was set at 2, the peptide length was set at 8–25, and the maximum variable modifications were set to carbamidomethyl on lysine and oxidation on methionine. 3. The precursor charges were set to 2 and 3, ions to 1 and 2, and ions to b, y, and p during the transition. The ion match tolerance was set to 0.02 Da, and the product ions were set from ion 3 to the final ion.

### 2.5 Establishment and estimation of prognostic signature

The RNA sequencing expression data were downloaded from the Cancer Genome Atlas (TCGA) dataset (https://portal.gdc.com) to compare these four verified DEPs between GC saliva and normal samples, including differential expression analysis and patient survival analysis. KM curves with the log-rank test were used to show the relationship between the expression of candidate genes and disease-free survival (DFS) in GC patients. All analysis methods and R packages were implemented using the R language. *Statistical significance was set at P < 0.05*.

## 3 Results

### 3.1 Saliva protein identification and quantification of GC

**FIGURE 1:**
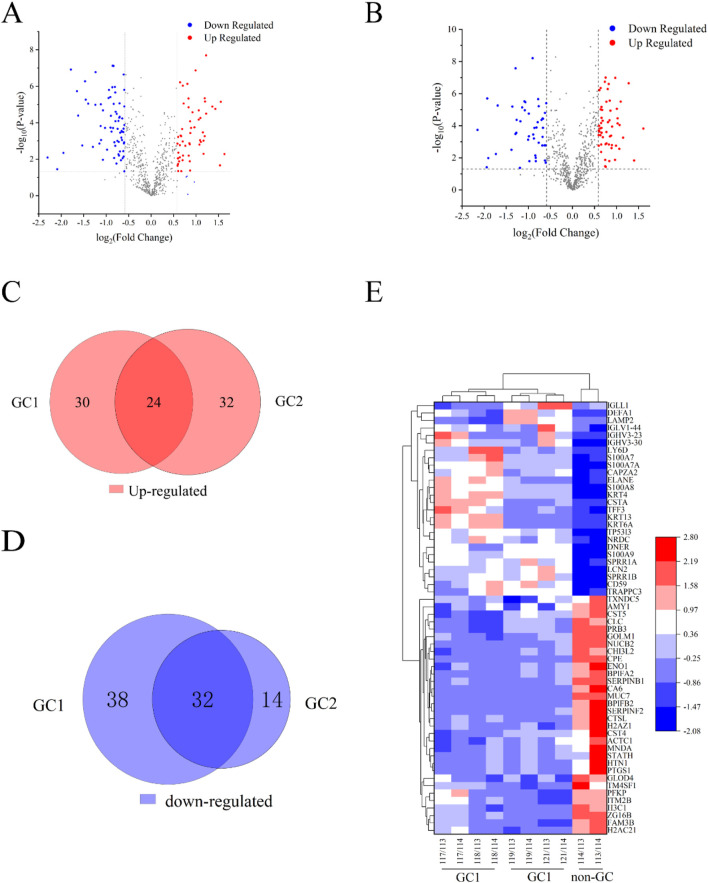
Results from quantitative proteomic analysis screened from iTRAQ. **(A)** Volcano plot of DEPs in the GC 1 group. **(B)** Volcano plot of DEPs in the GC 2 group. **(C)** Venn diagram of upregulated DEPs between GC 1 and GC 2. There were 24 overlapping DEPs between the two groups. **(D)** Venn diagram of downregulated DEPs between GC 1 and GC 2. **(E)** Cluster analysis of DEPs in the GC 1, GC 2, and non-GC groups. Red nodes represented significantly upregulated proteins with FC > 1.5 and *P* < 0.05. Blue nodes represented the significantly downregulated proteins with FC < 0.67 and *P* < 0.05. The gray nodes represent non-differentiated proteins.

In the biomarker discovery phase using iTRAQ, 36 saliva samples were analyzed, including the GC group 1 (*n =* 12), the GC group 2 (*n =* 13), and the non-GC group (*n =* 11). In the validation phase using PRM, 32 independent samples were analyzed (GC, *n =* 16; non-GC, *n =* 16). These group sizes were balanced for age and gender as shown in Tables 1 and 2. In this study, 671 salivary proteins were identified, each supported by at least one unique peptide and a confidence score of 20 or higher. A total of 124 or 102 proteins were significantly differentially expressed in GC groups 1 and 2, with *p-value* ≤ 0.05 and fold changes ≥1.500 or ≤0.657. The proteins in GC groups 1 and 2 are depicted in the volcano map shown in Figures 1A,B. A total of 56 overlapping DEPs between GC groups 1 and 2 were identified (Tables 1 and 2); 24 DEPs were overexpressed in GC groups 1 and 2 compared to the non-GC group, while 32 DEPs were underexpressed in GC groups 1 and 2 (Figures 1C,D). A clustering heat map for the subset of DEPs depicts the differential expression of salivary proteins that were found to be changed between the GC and the non-GC groups (Figure 1E).

### 3.2 Functional enrichment analysis of the DEPs in GC

**FIGURE 2:**
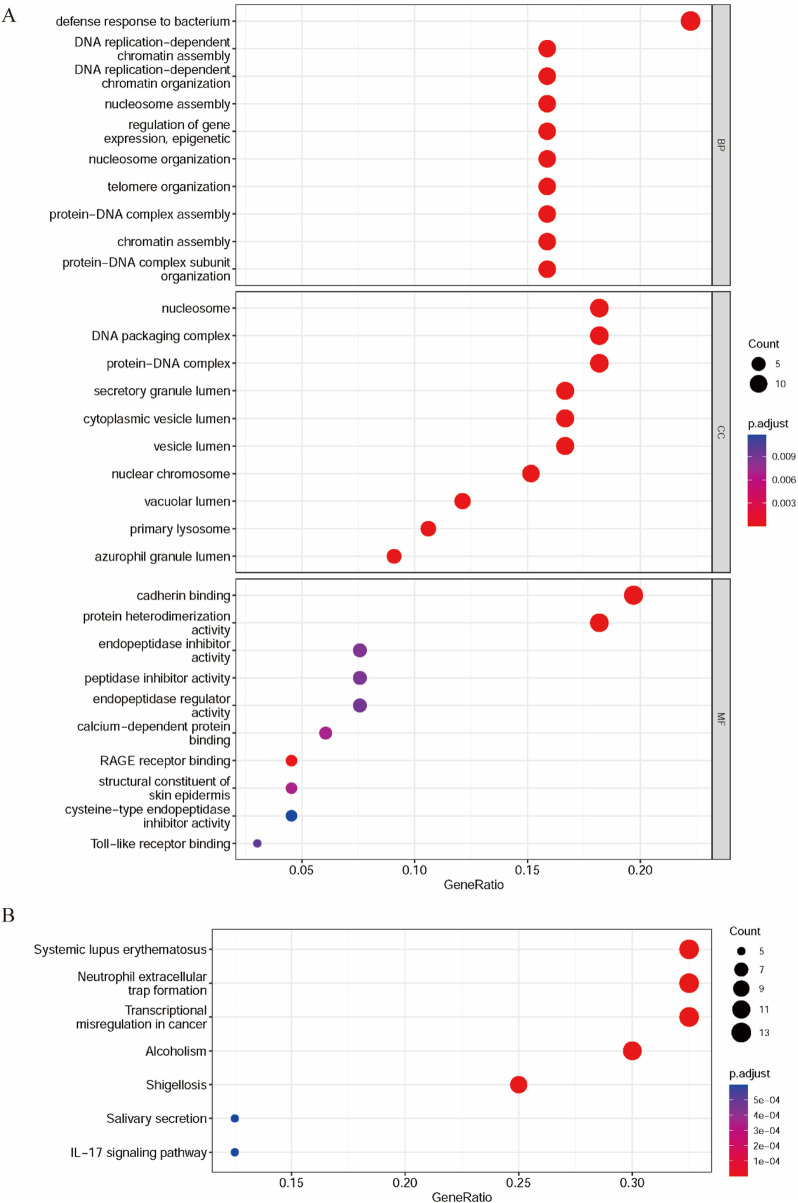
Functional enrichment analysis and PPI of DEPs in GC. **(A)** GO analysis of DEPs. **(B)** KEGG pathway of DEPs.

Bioinformatics analysis of DEPs can show potential interactions and discrepancies between proteins with reference to specific functionalities (Denny et al., 2018), reveal functional protein–protein interactions, and highlight functional differences across pathways. Therefore, the DEPs were subjected to enrichment analysis using the Gene Ontology (GO) classification and Kyoto Encyclopedia of Genes and Genomes (KEGG) pathway analysis. Biological process, cellular component, and molecular function are the three high-level categories used by the GO annotation to explain the biological function of proteins. A total of 65 biological process terms, 16 cellular component terms, and 12 molecular function terms were significantly enriched, with both adjusted p-values (p.adjust) and q-values less than 0.05. The top 10 terms in each category are presented in Figure 2A. Table 3 provides a detailed summary of the top enriched Gene Ontology (GO) terms and KEGG pathways, including the number of associated genes, adjusted p-values, and their biological relevance. These enrichments highlight the key molecular processes potentially involved in GC pathogenesis.

**TABLE 3:** Top GO and KEGG enrichment terms associated with differentially expressed salivary proteins in GC.

Category	Term name	Gene count	p.adjust	q-value	Description/pathway
BP	Defense response to bacteria	12	0.0012	0.002	Immune response to microbial stimuli
BP	Chromatin assembly	9	0.0035	0.004	Epigenetic regulation of gene expression
CC	Nucleosome	8	0.0009	0.001	Histone-associated DNA packaging
MF	Cadherin binding	11	0.0041	0.006	Cell–cell adhesion molecules
KEGG	Transcriptional misregulation in cancer	10	0.0028	0.004	Disruption of transcription factor pathways
KEGG	IL-17 signaling pathway	6	0.0053	0.006	Inflammatory signaling in the tumor microenvironment

Note: GO terms were grouped into biological processes (BP), cellular components (CC), and molecular functions (MF). Significance was based on adjusted p-values (p.adjust) and q-value thresholds <0.05.

According to the biological process classification, most of these proteins are utilized in defense response to bacteria, regulation of gene expression, epigenetic, protein–DNA complex assembly or organization, DNA replication-dependent chromatin assembly or organization, nucleosome assembly or organization, telomere or nucleosome organization, or chromatin assembly (Zhang et al., 2016). The results of the cellular component classification revealed that most of these DEPs were components of the nucleosome, DNA packaging complex, protein–DNA complex, secretory granule lumen, and cytoplasmic vesicle lumen. These DEPs were categorized by their molecular functions, including cadherin binding and protein heterodimerization activity. KEGG is a collection of databases for comprehending biological processes, such as metabolic pathways, biomolecular complexes, and biochemical reactions. The biological pathways of DEPs were identified using KEGG biological pathway enrichment analysis. The seven biological pathways with p. adjust<0.05 and q-value < 0.05 are shown in Figure 2B. We found that the genes of the DEPs were mainly enriched in systemic lupus erythematosus, neutrophil extracellular trap formation, transcriptional misregulation in cancer, alcoholism, shigellosis, IL-17 signaling pathway, and salivary secretion.

**FIGURE 3:**
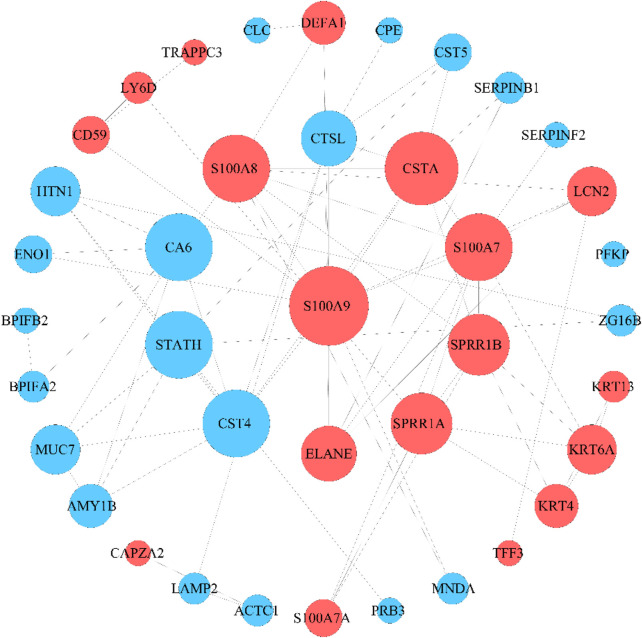
PPI network of DEPs. The size of the nodes was proportional to the degree of centrality determined by topology analysis. Upregulated proteins are marked in red, and downregulated proteins are marked in blue. The color and size of nodes are determined by the *p-value* or number of associated genes. The color from blue to red reflects the value of *P* from large to small, and the size, from small to large, reflects the number of associated genes from less to more.

The 56 DEPs were input into the STRING database, their relationship pairs were screened with interaction scores> 0.4, and a protein–protein interaction (PPI) network was constructed (Figure 3). Finally, 39 key DEPs were identified, of which 18 were significantly upregulated and 21 were significantly downregulated. These proteins are involved in cell adhesion, growth, proliferation, and apoptosis.

### 3.3 Verification of DEPs in GC via PRM

**FIGURE 4:**
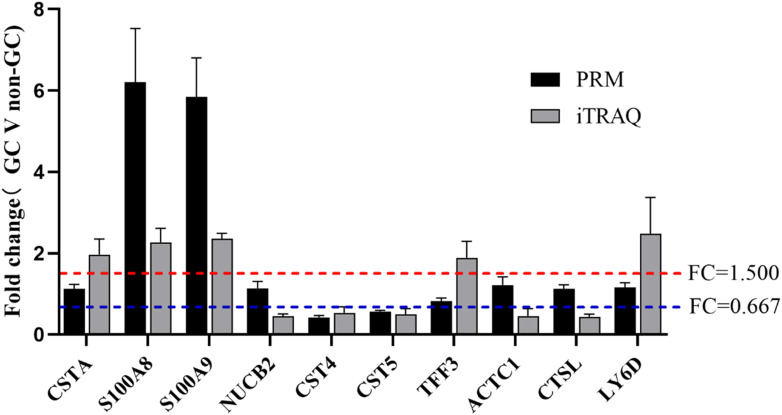
Comparison between the iTRAQ-based results and the PRM-based results.

**FIGURE 5:**
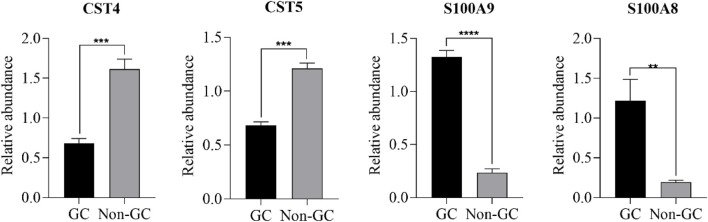
Comparison of protein expression between the GC and non-GC groups using PRM ( *P < 0.05, **P < 0.01, ***P < 0.001, and ****P < 0.0001).

To validate the iTRAQ findings, ten DEPs were selected based on their significance in GO, KEGG, and PPI analyses for further verification via PRM. These included S100A8, S100A9, CST4, CST5, and six additional genes. PRM analysis was conducted on 32 saliva samples (GC: *n =* 16; non-GC: *n =* 16). Of the 10 candidate proteins, four (S100A8, S100A9, CST4, and CST5) showed consistent expression trends between the iTRAQ and PRM datasets, with statistically significant differences (p < 0.05) (Figures 4, 5). The remaining six proteins failed PRM validation due to one or more of the following reasons: low peptide abundance in saliva, interference from co-eluting peptides, or poor signal-to-noise ratios during PRM detection. These technical limitations have likely reduced detection sensitivity for some candidates, highlighting the importance of optimizing PRM conditions for low-abundance salivary biomarkers.

Although formal ROC analysis was not performed in this pilot study due to the limited sample size, preliminary group comparisons by PRM revealed significant expression differences in S100A8, S100A9, CST4, and CST5 (*p* < 0.05), suggesting their potential diagnostic relevance. Future studies with larger cohorts will enable a robust evaluation of sensitivity, specificity, and classification accuracy using statistical or machine learning models (Table 4).

**TABLE 4:** Summary of differential expression and diagnostic potential of validated biomarkers (PRM phase).

Protein	Direction in GC	p-value (PRM)	Prior evidence (tissue/serum)	Diagnostic insight
S100A8	Upregulated	<0.001	Overexpressed in GC tissue	Promising
S100A9	Upregulated	<0.001	Overexpressed in GC tissue	Promising
CST4	Downregulated	<0.01	Upregulated in serum/tissue	Novel saliva pattern
CST5	Downregulated	<0.01	Upregulated in colorectal cancer (CRC) tissue	Novel saliva pattern

### 3.4 Establishment and estimation of the four genes’ prognostic signature

**FIGURE 6:**
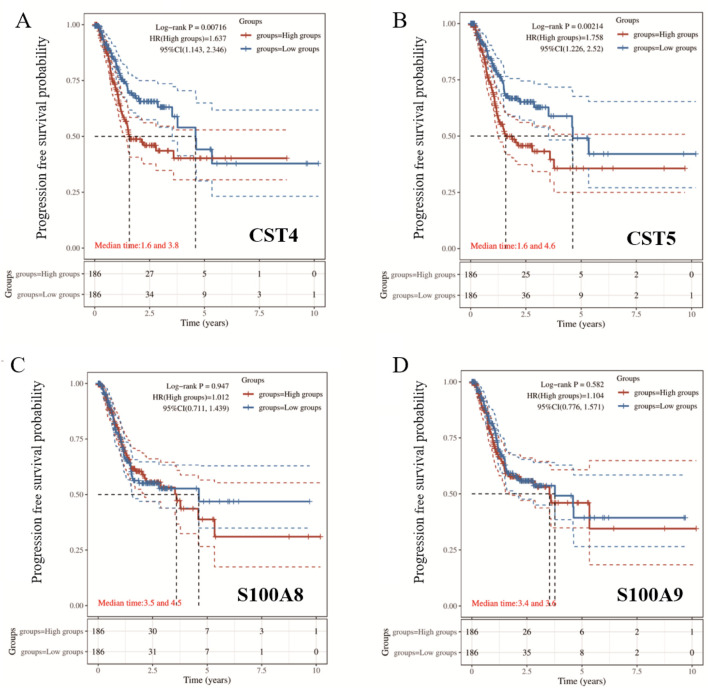
Kaplan–Meier survival analysis of **(A)** CST4, **(B)** CST5, **(C)** S100A8, and **(D)** S100A9. Survival curves compare high- and low-expression groups. Median survival times, hazard ratios (HR), and log-rank p-values are indicated in each panel.

The patients were stratified into high-risk and low-risk groups by the median risk score. KM survival analysis with the log-rank test was used to validate the multigene prognostic signature. HR > 1 indicates that the gene is a risk factor for poor progression-free survival (PFS), and HR < 1 indicates that the gene is a protective factor for good PFS. The KM survival curves showed that CST5 and CST4 were risk factors, and high CST5 and CST4 levels in cancer tissue were associated with worse PFS, compared with the low-risk group (*P* < 0.05) (Figure 6).

## 4 Discussion

Gastric cancer (GC) remains a leading cause of cancer-related mortality worldwide, largely due to late-stage diagnosis and the lack of effective early screening tools. In this study, we used iTRAQ and PRM-based proteomic analyses to identify salivary proteins associated with GC and discovered four differentially expressed proteins (S100A8, S100A9, CST4, and CST5) that were consistently altered in both analytical platforms.

Notably, we report for the first time that CST4 is downregulated in saliva samples from GC patients, a novel and unexpected finding, given that CST4 is typically overexpressed in GC tissue and serum. This inverse expression pattern suggests that saliva may reflect distinct molecular processes compared to other biofluids and highlights CST4 as a promising noninvasive biomarker for GC detection.

Cancer is the second major cause of higher mortality rates in the world and an important barrier to increasing life span in every country, placing a major economic burden on public health systems (Abbafati et al., 2020; Bray et al., 2021). Many cases of gastric cancer are diagnosed at an advanced stage due to low rates of early screening and limited treatment options. Specifically, GC prognosis is based on invasive procedures, such as upper digestive endoscopy, and traditional biomarkers for GC prognosis show low sensitivity and specificity. Therefore, it is urgent to find a less invasive but more accessible screening method for the diagnosis of GC.

We aimed to identify saliva proteins that changed between GC patients and healthy individuals using iTRAQ and PRM quantitative analysis and determine appropriate candidate proteins as new biomarkers related to GC. Our study identified 671 proteins with one or more unique peptide segments and scores ≥20 in this study. A total of 124 and 102 proteins showed statistically significant expression differences in GC group 1 and group 2, respectively. Hierarchical clustering analysis and Venn diagrams revealed that 58 saliva proteins were significantly altered in GC, including 24 upregulated and 23 downregulated proteins. These DEPs were implicated in several biological processes associated with gene expression regulation, epigenetics, and. antimicrobial humoral response (Wang et al., 2023).

Functional enrichment analysis revealed several pathways that are highly relevant to GC biology. For example, GO terms such as “chromatin assembly” and “regulation of gene expression” reflect epigenetic alterations commonly observed in GC pathogenesis. Enrichment in “cadherin binding” and “cell adhesion” points to mechanisms of tumor invasion and metastasis, as GC progression is often associated with epithelial–mesenchymal transition (EMT). KEGG analysis identified “transcriptional misregulation in cancer” and the “IL-17 signaling pathway” as key pathways associated with differentially expressed proteins. Dysregulation of transcriptional programs is a hallmark of cancer, and IL-17 signaling has been increasingly recognized for its role in gastric inflammation and tumorigenesis. Together, these enriched pathways suggest that salivary DEPs may reflect systemic biological changes relevant to GC development, including S100A7, S100A8, and S100A9. Not all of the DEPs detected by iTRAQ could be verified by PRM due to technical limitations. Using bioinformatics analysis, we selected 10 key DEPs for validation via PRM. Among these, four proteins, S100A8, S100A9, CST4, and CST5, showed consistent expression patterns in both iTRAQ and PRM analyses.

In most cancer cases, S100 protein dysregulation occurs, typically involving upregulation. It has been said that different carcinomas showed different S100 protein signatures (Bresnick et al., 2015; Allgöwer et al., 2020). So, these proteins are promising markers for the identification and prediction of staging of human tumors (Bresnick et al., 2015). While ten candidate proteins were selected for validation based on their functional relevance and differential expression patterns, only four proteins (S100A8, S100A9, CST4, and CST5) showed consistent and statistically significant expression trends between iTRAQ and PRM analyses. Several factors likely contributed to the lack of validation of the remaining six proteins. These include low peptide abundance in saliva, interference from background noise or co-eluting peptides, and technical limitations in PRM sensitivity and transition interference.

Quality control (Q.C.) procedures were implemented in the PRM analysis, including the use of pooled samples, internal standards, and Skyline software for retention time alignment and peak integration. Peptides that failed to meet the minimum signal-to-noise thresholds or reproducibility across replicates were excluded. The relative standard deviation (RSD) of the validated peptides across technical replicates remained below 15%, supporting the reliability of the four confirmed biomarkers. Further optimization of peptide selection, chromatographic conditions, and targeted transitions is necessary in future studies to improve validation rates. In iTRAQ analysis, we found that S100A7, S100A7A, S100A8, and S100A9 were upregulated in both GC groups 1 and 2. PRM analysis confirmed the upregulation of S100A8 and S100A9 in saliva from patients with gastric cancer. It has been reported that S100A8 and S100A9 levels may be a potential prognosticator of DFS in tumor patients; a high percentage of S100A8 and S100A9 means a low DFS (Koh et al., 2021). The normal range of calprotectin (S100A8/S100A9 heterodimer) in human serum is less than 1 μg/mL, but it increases in many types of cancers or inflammatory diseases (Shabani et al., 2018). Calprotectin has been documented as being overexpressed in neoplastic cancer cells and many other human tumor tissues or serum, such as nasopharyngeal carcinoma, GC, and bladder cancer (Yasar et al., 2017; Shabani et al., 2020; Xu et al., 2021). Highly concentrated calprotectin is cytotoxic and can induce apoptosis in AGS cell lines, the common type of gastric adenocarcinoma cell line, due to its effect on the Bax/Bcl-2 expression ratio and ability to inhibit ERK activation (Shabani et al., 2020). Upregulation of S100A7 protein has been predicted in cancer from several tissues (oral, esophagus, and breast) or serum (Dickinson et al., 2018; Mayama et al., 2018; Lu et al., 2021) with a strong correlation to poor prognosis. Upregulated S100A7 promotes cancer cell proliferation and migration through intracellular attachment to JAB1 as well as secretion and activation of RAGE receptors (Lu et al., 2021). Therefore, the expression of S100 family proteins, such as S100A8, S100A9, and S100A7, was associated with GC progression, which can be verified in GC saliva.

Recent studies linking cystatins (CSTs) to cancer have drawn increased interest. There is a CST superfamily of endogenous and reversible proteins that has an impact on controlling the excessive activity of cysteine peptidases in intracellular and extracellular environments (Breznik et al., 2019). Moreover, it is imperative to precisely maintain the right balance between CSTs and cysteine proteases because it is thought that their breakdown can result in the development of malignancies. A possible method for early identification of gastrointestinal cancer in patients could be serum CST4 detection (Cai et al., 2022). As a novel serum marker for gastrointestinal cancer, the positive detection rate of CST4 for gastrointestinal cancer is much higher than that of traditional markers such as CEA, CA199, CA125, and CA724, showing great superiority in sensitivity. CST4 is markedly upregulated in GC tissues, serum, or cells and is related to poor prognosis (OS) and progression-free survival (PFS) (Dou et al., 2018). CST4 overexpression promotes invasion and migration abilities of the GC cell lines MKN-45 and SGC-7901 *in vitro* and pulmonary metastasis *in vivo*, whereas silencing endogenous CST4 causes an opposite outcome (Zhang et al., 2017). CST5 is a proposed tumor suppressor induced by the p53 or vitamin D3 pathway in colorectal cancer (CRC) that suppresses tumor progression and metastasis. CST5 is identified as a significant mediator of tumor suppression by mediating mesenchymal-epithelial transition (MET) in CRC cells (Hünten and Hermeking, 2015). CST5 represses the expression of EMT inducers SNAI1, SNAI2, ZEB1, and ZEB2 and induces the expression of E-cadherin and other adhesion proteins. Furthermore, CST5 restricted migration and anchorage-independent growth, antagonized the Wnt/β-catenin signaling pathway, and suppressed c-MYC expression (Álvarez-Díaz et al., 2009). As a type of secreted protein, CST4 is secreted by the salivary and lacrimal glands, but its expression in the blood is lower. Gastric and intestinal tumor cells secrete CST4, which is transported to the blood, so the detection of serum CST4 has been well-defined as conducive to diagnosing some malignancies, mainly gastrointestinal tumors. In contrast to previous reports of CST4 and CST5 being upregulated in gastric cancer tissue and serum, our study found both proteins to be significantly downregulated in saliva samples from GC patients. This inverse pattern suggests a unique regulatory mechanism in salivary secretions that does not mirror systemic circulation. One possible explanation is that CST4 and CST5, both members of the cystatin family, are expressed and regulated differently in salivary glands compared to tumor cells or blood components. Local factors such as inflammation, microbial enzymatic activity, or altered secretory pathways in the salivary glands of GC patients may contribute to reduced salivary levels. Additionally, CST4/CST5 may be consumed or degraded more rapidly in the oral cavity environment of cancer patients due to shifts in oral microbiota or protease activity. These findings highlight the importance of validating biomarker behavior in biofluid-specific contexts and suggest that salivary expression patterns may reflect a combination of systemic and local physiological changes rather than simply echoing serum or tissue levels.

Currently, identifying new biomarkers of cancer has become a major target for cancer studies. These biomarkers are useful in diagnosis, monitoring, and therapeutic efficiency. In this study, we used iTRAQ and PRM-based quantitative proteomics to detect four saliva protein biomarkers of gastric cancer, including S100A8, S100A9, NUCB2, and CST4, which make them potential novel biomarkers for the noninvasive diagnosis of GC. These identified biomarkers can be definitively validated for GC detection, and it is a promising approach for screening GC patients and reducing endoscopies. Our study enhances the aspect of salivary diagnostics in the findings of other systemic diseases. The novelty of our study is an improvement in detection techniques. Although we faced some difficulties and limitations, such as a small sample size, more research is warranted to elucidate the diagnostic value of these biomarkers.

One of the primary limitations of this study is the relatively small cohort size (*n =* 68), with participants further divided into subgroups for discovery (iTRAQ) and validation (PRM) phases. This limited sample size may reduce the statistical power of our findings and increases the risk of false positives or overfitting, particularly in high-dimensional proteomic analyses. Additionally, the absence of external or multicenter validation limits the generalizability of our candidate biomarkers to broader clinical populations. Future studies involving larger, demographically diverse cohorts and prospective validation in independent datasets will be essential to confirm the diagnostic utility and robustness of these salivary protein biomarkers for gastric cancer.

This study has several limitations. First, the relatively small sample size (*n =* 68) may limit statistical power and increase the risk of type I and type II errors, particularly in high-dimensional proteomic analyses. This sample size also restricts subgroup analyses by cancer stage or histological subtype. Second, the absence of external or multicenter validation cohorts limits the generalizability of the findings to broader clinical populations. Future studies should include diverse cohorts and independent validations to confirm the diagnostic utility of these biomarkers. Third, although we used pooled samples and consistent protocols, potential batch effects during iTRAQ and PRM runs cannot be entirely ruled out. Variations in sample preparation, mass spectrometry conditions, or reagent lots could introduce technical variability. To minimize this, strict quality control procedures were followed, but further randomized sample processing and inter-batch normalization strategies are recommended in follow-up studies.

## 5 Conclusion

In this study, we employed iTRAQ and PRM-based quantitative proteomic analyses to investigate salivary protein profiles in gastric cancer (GC) patients compared with healthy controls. We identified a set of differentially expressed proteins (DEPs) with significant implications for GC pathology. Notably, four key proteins, S100A8, S100A9, CST4, and CST5, were validated as potential biomarkers through PRM analysis, with CST4 and CST5 exhibiting unique downregulation in saliva compared to tissue and blood, suggesting novel diagnostic insights. Our findings highlight the potential utility of saliva-based proteomics for the development of noninvasive, low-cost diagnostic biomarkers for GC, offering a promising avenue for early detection and monitoring of this malignancy. To facilitate clinical translation, future work should include large-scale, multicenter studies to validate the reproducibility and generalizability of these salivary biomarkers across diverse populations. Additionally, integrating multiple candidate proteins into diagnostic panels using machine learning or statistical modeling could increase classification accuracy and reduce false positives. Development of a saliva-based assay using these biomarkers may ultimately provide a cost-effective, noninvasive screening tool for early GC detection in clinical practice.

## Data Availability

The original contributions presented in the study are publicly available. This data can be found here: https://figshare.com/s/335c3a126a301765aed8.
